# β-globin gene cluster haplotypes in ethnic minority populations of southwest China

**DOI:** 10.1038/srep42909

**Published:** 2017-02-16

**Authors:** Hao Sun, Hongxian Liu, Kai Huang, Keqin Lin, Xiaoqin Huang, Jiayou Chu, Shaohui Ma, Zhaoqing Yang

**Affiliations:** 1 The Department of Medical Genetics, Institute of Medical Biology, Chinese Academy of Medical Sciences & Peking Union Medical College, 935 Jiaoling Road, Kunming 650118, China

## Abstract

The genetic diversity and relationships among ethnic minority populations of southwest China were investigated using seven polymorphic restriction enzyme sites in the β-globin gene cluster. The haplotypes of 1392 chromosomes from ten ethnic populations living in southwest China were determined. Linkage equilibrium and recombination hotspot were found between the 5′ sites and 3′ sites of the β-globin gene cluster. 5′ haplotypes 2 (+−−−), 6 (−++−+), 9 (−++++) and 3′ haplotype FW3 (−+) were the predominant haplotypes. Notably, haplotype 9 frequency was significantly high in the southwest populations, indicating their difference with other Chinese. The interpopulation differentiation of southwest Chinese minority populations is less than those in populations of northern China and other continents. Phylogenetic analysis shows that populations sharing same ethnic origin or language clustered to each other, indicating current β-globin cluster diversity in the Chinese populations reflects their ethnic origin and linguistic affiliations to a great extent. This study characterizes β-globin gene cluster haplotypes in southwest Chinese minorities for the first time, and reveals the genetic variability and affinity of these populations using β-globin cluster haplotype frequencies. The results suggest that ethnic origin plays an important role in shaping variations of the β-globin gene cluster in the southwestern ethnic populations of China.

According to the 2010 population census, excluding the Han, who forms the majority of the Chinese population, there are 55 other officially recognized ethnic minority populations accounting for 8.49% of the Chinese national population. More than 30 Chinese ethnic minorities inhabit in southwest China, most of them inhabit only in the region, such as Thai, Achang, Deang, and Tibetan. Due to the plateau and mountainous geographic features of the region, these populations have adapted the distinctive natural environment including climate, high altitude and epidemic infectious diseases, also experienced founder effect resulted from regional geographic isolation. Therefore, ethnic minority populations of southwest China present abundant diversity and distinction on their historic origins, languages, cultures, and genetic characteristics. Many studies have focused on understanding the genetic structures and relationships in these ethnic populations using microsatellite, mitochondrial DNA (mtDNA) and Y chromosome markers^1^,^2^,^3^. The evidence from these separate analyses concludes that the ancestors of East Asians originated in Africa and entered Asia from the southeast, but the migration of the ancient populations to the north of China and the strong south/north distinction in genetic patternation have been disputed^2^,^4^. In spite of the controversy, southwest China played an important role either as a passage of migration from Southeast Asia toward China or as an interface of ethnic mixture, and therefore impacted on the formation and diversity of Chinese ethnic populations. Furthermore, correlation between linguistic affinity and genetic diversity has been observed in southwest Chinese minorities^5^,^6^. However, the contributing genetic factors responsible for the diversities and affinities of these Chinese minorities remain unclear.

The value and power of β-globin gene cluster markers in resolving origins, migration and evolutionary relationships within human populations worldwide have been well demonstrated^7^,^8^,^9^,^10^,^11^,^12^. Seven neutral polymorphic restriction enzyme sites, especially the five sites within the 5′ region of the β-globin gene cluster, have been commonly employed to construct haplotypes for assessing genetic variation and the relationship between human populations. This method has revealed the origins of the β-globin gene mutations and the clinical implications of these haplotypes, including their links to heamoglobinopathies, such as β-thalassemia and sickle cell anemia. The distinct advantage of the β-globin gene cluster approach is that different populations from different studies are easily comparable since the same restriction sites and haplotypes have been widely used. Although many studies using the β-globin gene cluster haplotype have been carried out in a variety of populations in Africa, Europe, America and Asia, only a few northern Chinese ethnic populations have been analyzed^13^,^14^. In spite of their important roles in origin, migration and evolutionary history of Chinese ethnic populations, a large number of southwestern Chinese minority populations have not been investigated for β-globin gene cluster characteristics and haplotype variation. This lack of comparable data from southwestern minorities significantly restricts our understanding of Chinese ethnic diversity, differentiation and genetic relationships.

In the present study, we examine for the first time the allelic and haplotypic characteristics of the β-globin gene cluster in 10 ethnic minority populations, mainly from southwestern China. This study also integrated these results with those data previously published for Chinese and other world populations, and evaluated the genetic variability and relationship among the ethnic Chinese populations.

## Results

### β-globin gene cluster polymorphism in southwest Chinese ethnic minority groups

Table 1 presents the allelic frequencies detected for the seven polymorphic restriction sites of the β-globin gene cluster in the 10 minority populations from southwestern China. We found all restriction sites were polymorphic in the Hardy-Weinberg equilibrium with a few exception. We also found HincII 5′ ε and Hinf I 3′ β had the highest frequencies, while HincII 5′ Ψβ had the lowest frequency across the minority groups. This indicates that the distribution patterns of allelic frequencies were homogenous among the populations.

**Figure 1:**
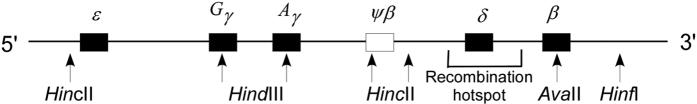
The location of the polymorphic restriction enzyme sites in the β-globin gene cluster.

We used a likelihood-ratio test of Arlequin software to evaluate linkage disequilibrium between a pair of loci in the studied populations from southwest China. Pairwise linkage disequilibrium (LD) were observed among the loci HincII 5′ ε, HindIII G γ, HindIII Aγ, HincII 5′ Ψβ and HincII 3′ Ψβ at the 5′ end of the β-globin gene cluster, as well as between AvaII β and Hinf I 3′ β loci at the 3′ end of the cluster. But LD was not observed between loci of the 3′and the 5′ ends of the cluster (Table 2). These suggest a recombination hotspot between the HincII 3′ Ψβ and AvaII β loci (Fig. 1), the haplotypes derived from these seven loci were divided into 5′ and 3′ sub-haplotypes according to the positions relative to the recombination hotspot.

### 5′ Haplotypes of the β-globin gene cluster in southwest Chinese ethnic groups

The haplotypes derived from the five polymorphic sites of the 5′ β-globin cluster from the 10 southwestern minority groups and other populations are reported in Table 3. Twenty-six of the 32 (2^5^) possible 5′ haplotypes were observed in the southwestern minority populations, but only haplotype 2, 5, 6 and 9 reached frequencies greater than 0.02. Haplotypes 25 (+++++) and 26 (++−+−) have never been described before this study. Seven haplotypes, 3, 17, 19, 20, 27, 28 and 29, were identified for the first time in Chinese populations with low frequencies as in other populations elsewhere. Haplotype 2 (+−−−−) was the most and 6 (−++−+) was the second most prevalent in the minorities of southwestern China with frequencies range of 0.570–0.779 and 0.035–0.174, respectively. The distribution patterns of common haplotypes 2, 6 and 5 in the southwestern minorities are generally consistent with that in other world populations. However, haplotype 2 was somewhat less frequent in Achang and Deang, and haplotype 5 was absent in Khmus. Achang participants had a significantly higher frequency of haplotype 4 (0.125), which is only slightly less frequent than that in African populations (0.152).

The distribution of the β-globin gene cluster haplotype showed geographic variation. The Chinese populations from the southwest and from north are distinguished by the distribution of haplotype 9 (−++++). Haplotype 9 is the third most prevalent haplotype with frequency of 2.2–8.5% in the southwest Chinese populations, but it is absent or very rare (<1%) in all other populations elsewhere (Table 3). On the contrary, common haplotype 5 (−+−++) in southwest China is less frequent than that in northern China. In addition, haplotypes 12 is observed in the ethnic minorities of Yunnan, but it is absent in other regions of China.

We also measured genetic variability of Chinese populations using heterozygousity and Gini-Simpson index (GSI). We found that heterozygousity and GSI of Achang, Deang and Khmus are much higher than that of all other Chinese populations except Oroqens from northern China, and are comparable with African. This suggests that the populations with longer history have higher levels of genetic variability.

### 3′ Haplotype analysis

We identified 3′ haplotypes based on the presence (+) and absence (−) of AvaII and Hinf I restriction sites (Fig. 1). We found gene framework (FW) 1–4 were polymorphic in all Chinese ethnic groups (Table 4). FW3 (−+) was the most common and FW4 (−−) was rare 3′ haplotypes in all ethnic Chinese populations examined. In general, the distribution patterns of FW1–4 among different Chinese ethnic populations were homogenous. In addition, we found different Thai subpopulations had the most frequent FW2, while Tibetan and northern Chinese Han subpopulations living in different regions had the highest FW3 haplotype frequencies. This suggests 3′ haplotype distribution pattern of these Chinese populations accords with their ethnic origin.

### Genetic diversity for the β-globin cluster in Chinese ethnic populations

The measures of genetic diversity in Chinese ethnic populations and other world populations are presented in Table 5. Overall, total heterozygosity (H_T_) of the southwestern Chinese population is 52.5%, of which 88% may be ascribed to genetic variation within populations. The level of heterozygosity observed in the southwestern Chinese is higher than that in northern Chinese, but the correction for gene differentiation coefficient (G_ST′_) is lower in southwest Chinese (2.5%) than in northern Chinese (3.7%), indicating less interpopulation differentiation among the southwest populations. The lowest G_ST′_ were observed in northern Chinese Han (0.5%) and Thai (0.7%) subpopulations, while high degree of interpopulation differentiation was observed in Tibetan (G_ST′_ = 1.2%), indicating genetic heterogeneity among different Tibetan subpopulations from different inhabitation regions. Likewise, when the northern Han were analyzed together with southern Han (Han), the G_ST′_ was significantly increased, suggesting high differentiation between south and north Chinese Han populations.

### Genetic relationship among Chinese populations

**Figure 2:**
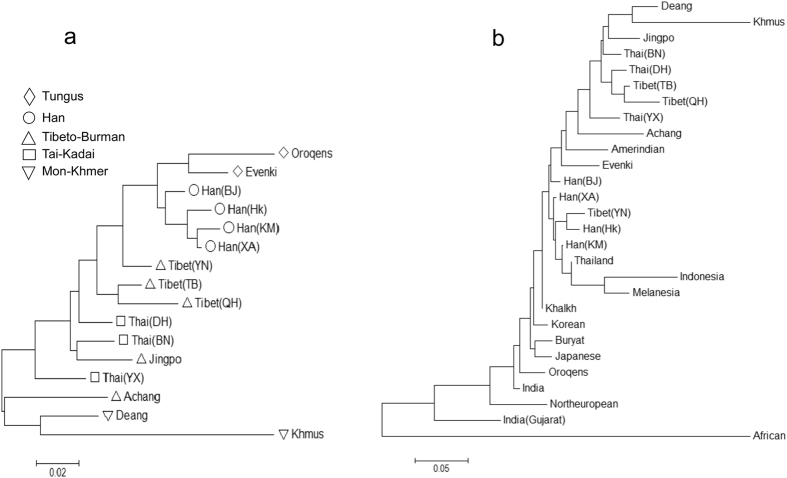
Unrooted Neighbor-joining tree showing genetic and linguistic affinities among the studied populations and other Chinese populations (**a**), and the genetic relationship among Asian, American, European and African based on 5′ haplotype frequencies of the beta-globin gene cluster (**b**).

Using pairwise Fst (F-statistics) and exact test of non-differentiation based on 5′ haplotype frequencies, the most significant differences and greatest genetic heterogeneity were observed in inter-ethnic comparisons of the Chinese populations (Supplementary Tables 1 and 2). Khmus and Deang were significantly different from other southwestern minority populations even though they live within a rather small geographic region. The phylogenetic relationships among the minorities of southwestern China and other populations are shown in the dendrogram Neighbor-joining (NJ) tree (Fig. 2), which is based on the matrix of genetic distances (DA) between the populations (Supplementary Table 3). The genetic affinities showed clear ethnic and linguistic patterns among Chinese populations (Fig. 2a). A southwest/northeast geographic pattern was observed as well, but the north/south division was not distinct. On a global scale, the ethnic minorities from southwestern China are closely clustered to other Chinese and Asian populations, but are far away from African and European populations. Amerindian from the American continent is clustered close to Chinese populations (Fig. 2b).

## Discussion

The β-globin gene cluster haplotypes have been largely shown as important and useful for investigating genetic variability, origin, migration and evolutionary relationships between populations worldwide. Our present study is the first report characterizing the β-globin gene cluster haplotypes in southwestern Chinese minorities, and reveals genetic variation and relationships in these ethnic populations. We found the allelic and haplotypic characterization of the β-globin gene cluster, and the significant differences in genetic variability, characteristics and distribution patterns of the haplotypes among the study populations. Our results demonstrate that current haplotypic variation of the β-globin gene cluster in Chinese ethnic populations mirrors their ethnic origin and linguistic classifications.

Our finding reveals the distribution characteristics of β-globin gene cluster haplotype in ethnic minority populations of southwest China. In the southwestern Chinese minorities, 5′ haplotype 2, 6, 9 and 5 are more prevalent overall, while haplotypes 12 and 4 are less common but not rare (Table 3). Haplotype 2 is the most common haplotype, and its distribution in southwestern China is consistent with that of the global pattern. Haplotype 6 was found to be the second most common haplotype in our study groups, which agree with its distribution pattern in Asian populations except in northern Chinese Han. Remarkably, we also found haplotype 9 is the third most frequent haplotype with frequencies of 2.2–8.5% in southwestern Chinese minorities (Table 3), which is much higher than that in northern Chinese populations (0–0.04) and other world populations^8^,^10^,^12^,^14^,^15^,^16^,^17^. Haplotype 9 is a characteristic haplotype across the southwestern Chinese minorities, suggesting gene flow and admixture among the adjoining populations within this geographic region. Moreover, in the Asian continent, haplotype 9 has only been reported in ethnic populations of China, to date. Comparable frequencies of haplotype 9 have been most clearly observed in native Americans that presumably migrated from Asia^10^,^16^,^18^. Our findings provide further evidence for Asian, probably Chinese, gene flow toward Native Americans.

The origin of β-globin gene cluster haplotypes in the populations of southwest China has not been explored. The haplotypes 2, 5 and 6 are considered primitive and first-order haplotypes as they are separated from each other by at least two genetic events, mutation or recombination. The origin of the rest of the haplotypes is derived from the first-order types by recombination^8^,^17^. Since at least two steps of genetic variations are required for conversion among haplotypes 2, 5 and 6, these three common haplotypes were likely present in the original populations settled in southwestern China. However, the prevalent haplotype 9 (−++++) in the populations of southwest China could be derived from all three common first-order haplotypes (2, 5, 6), most likely from haplotype 6 (−++−+) and 5 (−+−++), as only one conversion or mutation is needed at the HindIII G γ or HincII 5′ Ψβ sites, respectively. Nevertheless, there is not a one-to-one correspondence between pairs of ancestor haplotypes and their products of genetic conversion events^8^. Most second-order haplotypes are rare in the southwestern minorities; their origin could be attributed to genetic recombination.

Our results show linkage disequilibrium between the five sites within the 5′ region of the β-globin gene cluster and between the other two sites within the 3′ terminal, whereas linkage equilibrium was observed between 5′ and 3′ haplotypes as a result of the recombination hotspot between the two regions (Table 5, Fig. 1). These results confirm the presence of the recombination hotspot among the Chinese minority populations, consistent with findings in other populations^11^,^19^. As the polymorphic sites within 5′ haplotypes are in linkage disequilibrium and significantly associated to each other (Table 2), and the most common first-order haplotypes—presumably formed by a single round of recombination—were present in the study groups, our findings support the previous hypothesis that the rate of recombination within the 5′ haplotypes is not particularly rapid^11^,^19^. Therefore, the most common haplotypes 2, 6, 5 in southwestern Chinese minority populations would not be the result of recent recombination events, while it remains unclear if the same can be inferred for the common second-order haplotype 9.

5′ haplotypes provide information on microevolutionary processes, while 3′ haplotypes reveal the ancestral origins of populations and can be used to trace the origin of β-globin gene mutations^20^,^21^,^22^. Southwestern China is a region with endemic malaria and thalassemia, with the highest frequencies of hemoglobin E (β^E^, HBB codon 26 G > A) reported in the Achang, Deang and Jingpo minority populations living in the region^23^,^24^. The β^E^ gene was found exclusively linked to haplotype 9 and FW2 in southwestern Chinese minorities in our previous study^24^. But wide type *HBB* gene β^A^ (*HbAA*) was linked to 5′ haplotypes 2, 6, 9, 5 (Table 3) and all gene frameworks, mostly to FW3 (−+)in the populations (Table 4). The distribution pattern of the 3′ haplotype frameworks is homogeneous in southwestern minorities and similar to that in southeastern Asian populations^21^,^25^, which indicates a common origin in these Asian populations. More importantly, our results found that haplotype 9 is a characteristic type across all of the southwestern minority groups, meaning it must have been present in very early colonies of these populations. The findings on β^A^ linked haplotypes in this study provide additional information for inferring evolution of the β^E^ mutation. We speculate that the predominant β^E^ genes in southwestern minorities occurred on a common haplotype 9 bearing chromosomal background and spread into different populations through the adjoining effect. The haplotype 9 linked β^E^ is unlikely to form from haplotype 2 linked β^E^ through recombination events in the respective populations.

It is well known that high heterozygosity is attributed to long population histories or interpopulation gene flow. In the present study, haplotypic heterozygosity, GSI, considering both frequency and number of haplotypes, and number of effective haplotypes (Ne) in the Achang, Deang and Khmus from southwestern China was found to be much greater than that of other Chinese populations with the exception of Oroqens (Table 3). Deang is one of the oldest original populations, having lived in the region for more than 2000 years; Khmus is another older aborigine population in the same region as well-the higher heterozygosities reflect their longer ethnic history. As Achang originated from an ancient Di-qiang population living in the Qinghai-Tibet Plateau of China, migration and admixture are expected. The higher heterozygosity of Achang could mirror the evolutionary action of both ethnic history and gene flow. Our findings provide more genetic evidence for interpreting the history and migration of southwestern Chinese minorities.

Our study explores how ethnic minority populations of southwest China are related to each other and other populations. Our findings demonstrate that genetic affinities among ethnic Chinese populations show ethnic and linguistic patterns. Some studies using microsatellite and mitochondrial DNA (mtDNA) markers have found distinct genetic divergence between southern and northern Chinese populations, and have argued that northern populations are derived from southern Chinese populations^1^,^2^. Whereas, other studies found that DNA markers did not support the south/north division but rather suggest simple distance isolation^4^. In addition, the correlation between genetic diversity and linguistic affinity in Chinese ethnic groups was demonstrated by autosomal microsatellite markers^5^,^6^. By using the β-globin cluster markers, our study does not support the distinct south/north geographic division found in other studies using microsatellite, Y Chromosomal STR and mtDNA markers^1^,^2^,^3^, but tends to support the hypothesis that DNA marker patterns suggest simple isolation by geographic distance^4^. Alternatively, it is possible that the β-globin cluster markers and the other genetic markers may have evolved differently in these populations.

When genetic relationships between world populations were analyzed using haplotypic frequencies, the majority of Chinese ethnic groups were clustered together and close to other Asia and Amerindian populations as expected (Fig. 2b). As a result of the limitations in the number of examined populations and sample sizes, the unrooted polygenetic tree may only represent the genetic affinities but not evolutionary relationships among the populations. This study reveals genetic relationships of southwestern minorities of China using the β-globin gene cluster markers, for the first time, and provides new evidence supporting the consistency of genetic and linguistic evolution in Chinese populations. Moreover, our findings on the characteristic haplotype 9 distribution and phylogenetic relationship among populations strongly support the notion that Asian, and most likely Chinese, gene flow migrated toward native American populations^9^,^10^,^16^,^26^.

In conclusion, the β-globin haplotype is useful for elucidating genetic variation, affinity and ethnic origin of human populations. Here we have shown that the distribution of β-globin haplotypes is significantly heterogeneous in minority populations of southwest China, the distribution pattern is significantly different with that of populations in other regions of China. Moreover, we have demonstrated that the genetic affinity of Chinese population show ethnic and linguistic patterns. The genetic heterogeneity and differentiation presented in the southwest Chineses ethnic populations deepen our understanding of their ethnic history and gene flow. The diversity of the β-globin gene cluster in Chinese populations is mainly attributed to ethnic origin. Meanwhile, admixture, geographic isolation and genetic recombination are important factors accounting for the genetic variations observed. Overall, our findings provide new comparable data for revealing genetic diversity and the relationships of Chinese populations, and once again, and show that the β-globin gene cluster can provide a large amount of substantial information on elucidating human history and evolution.

## Materials and Methods

### Studied populations

**Figure 3:**
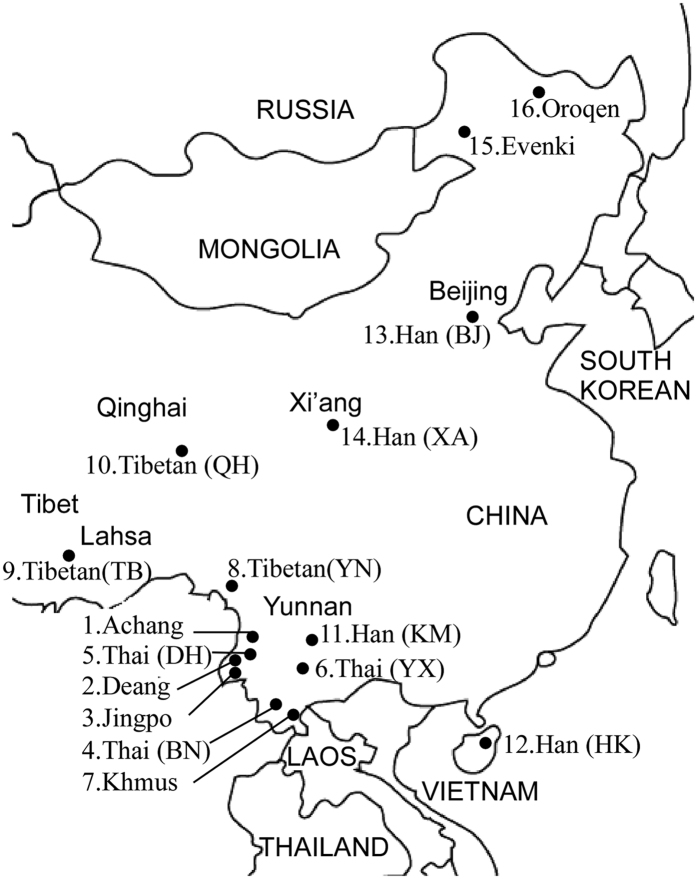
Outline map of Greater China indicating the geographic locations of Chinese ethnic populations sampled in present and previous studies. Details of the populations are presented in Table 6. The map was created using Canvas Software version 11, ACD Systems of America, Inc. Seattle, WA, USA. www.acdsystems.com.

Ten ethnic minority groups belonging to six Chinese nationalities: Achang, Deang, Khmus (officially recognized as a subpopulation of Bulang nationality), Jingpo, Thai (named Dai in Chinese) and Tibetan from south-to-west China regions including Yunnan, Tibet and Qinghai provinces, were studied. The chosen groups well represent ethnic populations of southwest China as they inhabit only in the region, with different historic origins and cultures. The sample size was chosen to satisfy the needs of genetic statistics. It varied with size and sample availability of the different ethnic groups. The sampling, geographic location and linguistic affiliation of the populations are presented in Fig. 3 and Table 6. All experiments and methods were approved and in accordance with the Ethics Committee of the Institute of Medical Biology, Chinese Academy of Medical Sciences. Unrelated healthy individuals from different minority populations were randomly selected. Informed consent was obtained from all subjects. Individual information on ethnic identification, ancestry and migration history were recorded to ensure the representativeness of their own minority communities. Genomic DNA was extracted from peripheral blood samples collected using anticoagulant sodium citrate. Carriers of the β-globin gene (*HBB*) mutations were excluded based on hematologic and molecular genetics analyses for thalassemia. A total of 1392 chromosomes with normal β-globin genotype *HbAA* (β^A^) from 696 individuals were examined in the present study.

### Genotyping of the β-globin gene cluster

There are seven polymorphic restriction sites in the β-globin gene cluster: HincII 5′ ε, HindIII G γ, HindIII Aγ, HincII 5′ Ψβ, HincII 3′ Ψβ, AvaII β and Hinf I 3′ β (Fig. 1); these sites were genotyped using polymerase chain reaction followed by restriction fragment length polymorphism (PCR-RFLP) protocols as previously described with a few modification^27^,^28^. The genotypes were recorded as “+“ or “−“ according to the presence or absence of the respective restriction enzyme sites.

### Haplotype analysis and statistical analysis

Allele frequencies for different restriction sites were calculated using a direct counting method. The Hardy-Weinberg equilibrium, estimation of haplotype frequencies of the β-globin gene cluster, linkage disequilibrium between pairs of loci, pairwise Fst (F-statistics) and the exact test of population differentiation, were evaluated using the population genetics analysis software Arlequin V3.5.2^29^,^30^. The genetic distance and phylogenetic analysis program DISPAN (http://www.personal.psu.edu/nxm2/dispan2.htm, copyright 1993 by Tatsuya Ota and the Pennsylvania State University) was used to measure genetic diversity parameters H_T_ (the average heterozygosity for the entire population), Hs (the average heterozygosity within populations) and G_ST_ (gene differentiation coefficient). The haplotype frequencies in the present study were integrated with those from other populations in China and around the world using data from previous reports^8^,^10^,^11^,^13^,^14^,^15^,^17^,^20^,^31^. The matrix of DA genetic distances between populations was calculated using the haplotype frequencies, and phylogenetic trees were constructed using DA distances through the DISPAN program. G_ST′_ was used as a correction of G_ST_ affected by the number of examined populations^16^. Genetic diversity was also measured by the Gini-Simpson index (GSI) and the effective number of haplotypes (Ne) as previously described^8^.

## Additional Information

**How to cite this article**: Sun, H. *et al*. β-globin gene cluster haplotypes in ethnic minority populations of southwest China. *Sci. Rep.* **7**, 42909; doi: 10.1038/srep42909 (2017).

**Publisher's note:** Springer Nature remains neutral with regard to jurisdictional claims in published maps and institutional affiliations.

## Supplementary Material

Supplementary Table

## Figures and Tables

**Table 1:** Allele frequencies for the presence (+) of the seven restriction loci of the β-globin cluster in the ethnic minorities of southwest China.

	Achang	Deang	Jingpo	Khmus	Thai (DH)	Thai (BN)	Thai (YX)	Tibetan (YN)	Tibetan (TB)	Tibetan (QH)
Chromosomes	104	166	164	140	268	144	136	92	96	82
HincII 5′ ε	0.625	0.693	0.762	0.693	0.813	0.764	0.794	0.739	0.750	0.683
HindIII G γ	0.413	0.373*	0.232	0.129	0.198	0.271	0.228	0.261	0.260	0.329
HindIII Aγ	0.154	0.169	0.183	0.164	0.160	0.188	0.140	0.196	0.219	0.305
HincII 5′ Ψβ	0.135	0.181	0.146	0.093	0.108	0.111	0.147	0.087	0.146	0.134
HincII 3′ Ψβ	0.394	0.295	0.262	0.236	0.205	0.222	0.213	0.261	0.260	0.317
AvaII β	0.529	0.440	0.537*	0.536	0.575	0.556	0.551	0.435	0.427	0.341
Hinf I 3′ β	0.683	0.735	0.713	0.721*	0.582	0.625	0.588	0.750	0.771	0.817

^*^Hardy-Weinberg disequilibrium (p < 0.05).

**Table 2:** Pairwise linkage disequilibrium (LD) test for seven polymorphic loci of the β-globin cluster in the southwest Chinese ethnic minority population.

Locus	HincII 5′ε	HindIII G γ	HindIII Aγ	HincII 5′Ψβ	HincII 3′Ψβ	AvaII β	Hinf I 3′ β
HincII 5′ ε		−897.986	−856.781	−925.626	−828.596	−1336.707	−1278.332
HindIII G γ	0		−909.472	−918.317	−904.678	−1340.644	−1284.383
HindIII Aγ	0	0		−878.712	−864.688	−1239.161	−1182.412
HincII 5′ Ψβ	0	0	0		−917.026	−1167.372	−1109.937
HincII 3′ Ψβ	0	0	0	0		−1345.222	−1288.511
AvaII β	**0.231**	**0.437**	**0.488**	**0.802**	**0.561**		−1241.530
Hinf I 3′ β	0.004	0.070	0.042	0.028	0.049	0	

The values above the diagonal are likelihood of LD (LnLHood) obtained by applying the EM algorithm to estimate haplotype frequencies, and the values below the diagonal are corresponding p-values of the likelihood-ratio test (d.f. = 1. Significance p-value = 0.05; number of permutations = 1000). Bold indicates the pairwise linkage equilibrium (p > 0.05 in the linkage disequilibrium test).

**Table 3:** 5′ haplotype frequencies of the β-globin cluster in the minorities from Southwest China, other Chinese populations and world populations.

Population		Thai (DH)	Thai (BN)	Thai (YX)	Jingpo	Achang	Deang	Khmus	Tibetan (YN)	Tibetan (TB)	Tibetan (QH)	Han^b^ (KM)	Han^b^ (XA)	Han^b^ (BJ)	Han^c^ (HK)	Evenki^b^	Oroqen^b^	Euro- Pean^d^	Amer- Indian^e^	African^d^
Chromosomes		268	144	136	164	104	166	140	92	96	82	240	254	226	110	228	162	258	424	79
5′- Haplotype^a^																				
1	−−−−−			0.015			0.007	0.0790		0.011		0.012				0.005	0.020		0.026	
2	+−−−−	0.779	0.707	0.717	0.700	0.566	0.570	0.607	0.739	0.729	0.659	0.763	0.737	0.686	0.809	0.669	0.572	0.609	0.728	0.063
3	−−−−+							0.036										0.008	0.022	0.532
4	−+−−+	0.012	0.008	0.008		0.125	0.048	0.021				0.008	0.008	0.013		0.019		0.004	0.022	0.152
5	−+−++	0.021	0.041	0.046	0.032	0.096	0.072		0.065	0.042	0.012	0.141	0.149	0.134	0.064	0.079	0.129	0.233	0.031	0.139
6	−++−+	0.078	0.103	0.065	0.084	0.095	0.078	0.036	0.174	0.115	0.183	0.067	0.087	0.118	0.1	0.099	0.178	0.112	0.110	0.025
7	−++−−	0.004	0.014					0.007			0.012		0.004	0.004			0.012	0.027	0.005	
8	+−−++				0.006											0.009				
9	−++++	0.064	0.049	0.049	0.079	0.038	0.057	0.036	0.022	0.083	0.085			0.005		0.004				
10	++−++	0.004		0.012	0.006		0.006							0.004		0.028	0.013	0.004		
11	−−−++			0.008	0.017			0.007			0.012	0.008	0.008	0.009	0.027	0.009	0.031			
12	++−−−		0.028	0.038	0.019	0.040	0.071	0.021					0.004			0.009	0.013		0.015	
13	+−−−+	0.011	0.014		0.024		0.006	0.029					0.004	0.014		0.005			0.027	0.038
14	++−−+					0.019											0.006		0.002	0.013
15	+++−+			0.010							0.012					0.042	0.025			0.025
16	−+−−−	0.004	0.007		0.006		0.018							0.005		0.003		0.004	0.012	0.013
17	+−+−−		0.008		0.006															
18	−+−+−		0.007		0.006						0.012			0.007		0.006				
19	+−+++						0.006													
20	−−+−+	0.004			0.014	0.020	0.021	0.071												
23	+++−−															0.009				
24	+−−+−	0.008		0.016			0.017									0.005				
25	+++++	0.011	0.007					0.036		0.0211	0.012									
26	++−+−						0.017													
27	−+++−		0.007				0.006													
28	−−+++			0.016																
29	−−++−							0.007												
Number of haplotype	12	13	12	13	8	15	14	4	6	9	6	8	11	4	16	10	8	12	9	
Heterozygousity	0.384	0.487	0.478	0.497	0.648	0.656	0.618	0.423	0.451	0.531	0.395	0.429	0.499	0.334	0.535	0.626	0.564	0.456	0.676	
GSI	0.382	0.484	0.474	0.493	0.642	0.652	0.613	0.419	0.446	0.525	0.393	0.427	0.497	0.331	0.533	0.622	0.561	0.455	0.668	
Ne	1.618	1.937	1.902	1.975	2.791	2.871	2.585	1.766	1.807	2.104	1.648	1.744	1.988	1.494	2.141	2.645	2.280	1.834	3.007	

^a^The nomenclature for haplotypes 1–24 is reported as in Long *et al*. and Shimizu *et al*.^8^,^14^, while designation of haplotypes 25–29 is given arbitrarily.

^b^Shimizu *et al*.^14^.

^c^Cheng *et al*. and Chan *et al*.^20^,^31^.

^d^Long *et al*.^8^.

^e^Callegari-Jacques *et al*.^10^.

**Table 4:** Frequencies of the β-globin gene cluster frameworks (FW, AvaII β-Hinf 3′) in Chinese ethnic populations.

population^a^	FW1 (++)	FW2 (+−)	FW3 (−+)	FW4 (−−)	reference
Achang	0.232	0.296	0.450	0.021	Present study
Deang	0.196	0.243	0.539	0.022	
Jingpo	0.282	0.255	0.432	0.032	
Thai (BN)	0.214	0.341	0.411	0.034	
Thai (DH)	0.157	0.418	0.425	0	
Thai (YX)	0.165	0.386	0.423	0.026	
Khmus	0.275	0.261	0.447	0.017	
Tibetan (YN)	0.185	0.250	0.565	0	
Tibetan (TB)	0.198	0.230	0.573	0	
Tibetan (QH)	0.200	0.141	0.617	0.041	
Han (BJ)	0.194	0.234	0.562	0.009	Shimizu. *et al*.^13^,^14^
Han (XA)	0.204	0.250	0.533	0.014	
Han (KM)	0.128	0.222	0.655	0.014	
Evenki	0.190	0.275	0.556	0	
Oroqen	0.160	0.189	0.649	0	

^a^Details of populations are described in Table 6.

**Table 5:** Genetic diversity for the β-globin gene cluster in world populations.

Population	No. of sub-populations	H_T_	H_s_	D_st_	D_m_	G_ST_	G_ST′_
Thai^a^	3	0.449	0.447	0.002	0.003	0.005	0.007
Tibetan^a^	3	0.467	0.463	0.004	0.006	0.008	0.012
Northern Han^b^	3	0.441	0.439	0.002	0.002	0.004	0.005
Han^c^	4	0.416	0.412	0.004	0.005	0.009	0.013
Southwestern Chinese^a^	10	0.525	0.513	0.012	0.013	0.022	0.025
Northern Chinese^b^	5	0.476	0.462	0.014	0.018	0.030	0.037
Chinese^d^	16	0.509	0.496	0.013	0.014	0.025	0.027
Asian^e^	24	0.532	0.508	0.024	0.025	0.045	0.047
Americans^f^	31	0.413	0.383	0.030	0.031	0.073	0.075
European^f^	5	0.622	0.588	0.034	0.043	0.055	0.067
African^f^	6	0.715	0.651	0.064	0.077	0.090	0.106

^a^The present study.

^b^Shimizu *et al*.^14^.

^c^Shimizu *et al*.^14^, Cheng *et al*.^31^, and Chan *et al*.^20^.

^d^The present study and Shimizu *et al*.^13^,^14^.

^e^Reference (number of populations): The present study (10), Shimizu *et al*. (9)^13^,^14^, Long *et al*. (2)^8^, Chen *et al*. (1)^17^, Aggarwal *et al*. (1)^11^, Chen and Chan *et al*. (1)^20^,^31^.

^f^Callegari-Jacques SM *et al*.^10^.

**Table 6:** Sample information of Chinese ethnic populations examined in the present study and previous reports.

Populations	No. of Chromosomes	Linguistic family, subfamily	Location (City, Province)	Geographic classification	reference
1. Achang	104	Sino-Tibetan, Tibeto-Burman	Lianghe, Yunnan	southwest	Present study
2. Deang	166	Austro-Asiatic, Mon-Khmer	Mangshi, Yunnan	southwest	
3. Jingpo	164	Sino-Tibetan, Tibeto-Burman	Mangshi, Yunnan	southwest	
4. Thai (BN)	144	Daic, Tai-Kadai,	Jinghong, Yunnan	southwest	
5. Thai (DH)	268	Daic, Tai-Kadai	Mangshi, Yunnan	southwest	
6. Thai (YX)	136	Daic, Tai-Kadai,	Yuxi, Yunnan	southwest	
7. Khmus	140	Austro-Asiatic, Mon-Khmer	Menhai, Yunnan	southwest	
8. Tibetan (YN)	92	Sino-Tibetan, Tibeto-Burman	Zhongdian, Yunnan	southwest	
9. Tibetan (TB)	96	Sino-Tibetan, Tibeto-Burman	Lahsa, Tibet	southwest	
10. Tibetan (QH)	82	Sino-Tibetan, Tibeto-Burman	Guinan, Qinghai	southwest	
11. Han (KM)	240	Sino-Tibetan, Chinese	Kunming, Yunnan	south origin	[14]
12. Han (HK)	110	Sino-Tibetan, Chinese	HongKong	south	[20, 31]
13. Han (BJ)	226	Sino-Tibetan, Chinese	Beijing	north	[14]
14. Han (XA)	254	Sino-Tibetan, Chinese	Xi’ang, Shangxi	north	[14]
15. Evenki	228	Altaic, Tungus	Hailar, Inner Mongolia	north	[13]
16. Oroqen	162	Altaic, Tungus	Heihe, Heilongjiang	north	[13]
