# Supplementary material for: β-globin gene cluster haplotypes in ethnic minority populations of southwest China
Source: Sci Rep. 2017 Feb 16;7:42909. doi: 10.1038/srep42909 (PMC5311981; doi:10.1038/srep42909)
Supplement: Supplementary Table [file srep42909-s1.doc]

**β-globin gene cluster haplotypes in ethnic minority populations of southwest China**

Hao Sun*, Hongxian Liu*, Kai Huang, Keqin Lin, Xiaoqin Huang, Jiayou Chu, Shaohui Ma, Zhaoqing Yang

The Department of Medical Genetics, Institute of Medical Biology, Chinese Academy of Medical Sciences & Peking Union Medical College, 935 Jiaoling Road, Kunming 650118, China.

*These authors contributed equally to this work.

Correspondence and requests for materials should be addressed to S.M (email: shouhuima@imbcams.com.cn) or Z.Y (email: zyang@imbcams.com.cn)

**Supplementary Table 1.** Pairwise FSTs in Chinese ethnic populations. (Significance level=0.05)

| population^a^ | Thai(DH) | Jingpo | Deang | Thai(BN) | Thai(YX) | Achang | Tibetan(QH) | Khmus | Tibetan(YN) | Tibetan(TB) | Han(HK) | Han(BJ) | Han(XA) | Han(KM) | Oroqen |  |
| --- | --- | --- | --- | --- | --- | --- | --- | --- | --- | --- | --- | --- | --- | --- | --- | --- |
| Thai(DH) | * |  |  |  |  |  |  |  |  |  |  |  |  |  |  |  |
| Jingpo | 0.099 | * |  |  |  |  |  |  |  |  |  |  |  |  |  |  |
| Deang | 0.000 | 0.009 | * |  |  |  |  |  |  |  |  |  |  |  |  |  |
| Thai(BN) | 0.099 | 0.847 | 0.018 | * |  |  |  |  |  |  |  |  |  |  |  |  |
| Thai(YX) | 0.216 | 0.613 | 0.000 | 0.847 | * |  |  |  |  |  |  |  |  |  |  |  |
| Achang | 0.000 | 0.009 | 0.405 | 0.000 | 0.000 | * |  |  |  |  |  |  |  |  |  |  |
| Tibetan(QH) | 0.000 | 0.225 | 0.072 | 0.207 | 0.090 | 0.009 | * |  |  |  |  |  |  |  |  |  |
| Khmus | 0.000 | 0.000 | 0.000 | 0.000 | 0.018 | 0.009 | 0.018 | * |  |  |  |  |  |  |  |  |
| Tibetan(YN) | 0.063 | 0.108 | 0.000 | 0.315 | 0.126 | 0.000 | 0.171 | 0.000 | * |  |  |  |  |  |  |  |
| Tibetan(TB) | 0.414 | 0.667 | 0.000 | 0.748 | 0.604 | 0.000 | 0.342 | 0.000 | 0.369 | * |  |  |  |  |  |  |
| Han(HK) | 0.144 | 0.036 | 0.000 | 0.072 | 0.018 | 0.000 | 0.018 | 0.000 | 0.225 | 0.126 | * |  |  |  |  |  |
| Han(BJ) | 0.000 | 0.000 | 0.000 | 0.135 | 0.054 | 0.000 | 0.027 | 0.000 | 0.162 | 0.063 | 0.009 | * |  |  |  |  |
| Han(XA) | 0.000 | 0.018 | 0.000 | 0.027 | 0.018 | 0.000 | 0.000 | 0.000 | 0.081 | 0.018 | 0.108 | 0.324 | * |  |  |  |
| Han(KM) | 0.000 | 0.000 | 0.000 | 0.009 | 0.018 | 0.000 | 0.000 | 0.000 | 0.018 | 0.072 | 0.081 | 0.099 | 0.676 | * |  |  |
| Oroqen | 0.000 | 0.000 | 0.018 | 0.009 | 0.000 | 0.054 | 0.027 | 0.000 | 0.036 | 0.000 | 0.000 | 0.027 | 0.000 | 0.000 | * |  |
| Evenki | 0.000 | 0.063 | 0.000 | 0.162 | 0.171 | 0.018 | 0.081 | 0.000 | 0.225 | 0.072 | 0.009 | 0.225 | 0.045 | 0.000 | 0.000 | * |

^a^ Details of populations are described in Table 6.

**Supplementary Table 2**. Exact test of non-differentiation in Chinese ethnic populations (significance Level=0.05)

| population^a^ | Thai(DH) | Thai(BN) | Thai(YX) | Jingpo | Achang | Deang | Khmus | Tibetan(YN) | Tibetan(TB) | Tibetan(QH) | Han(BJ) | Han(XA) | Han(KM) | Han(HK) | Evenki |
| --- | --- | --- | --- | --- | --- | --- | --- | --- | --- | --- | --- | --- | --- | --- | --- |
| Thai(DH) |  |  |  |  |  |  |  |  |  |  |  |  |  |  |  |
| Thai(BN) | 0.115 |  |  |  |  |  |  |  |  |  |  |  |  |  |  |
| Thai(YX) | 0.005 | 0.371 |  |  |  |  |  |  |  |  |  |  |  |  |  |
| Jingpo | 0.041 | 0.675 | 0.181 |  |  |  |  |  |  |  |  |  |  |  |  |
| Achang | 0.000 | 0.000 | 0.000 | 0.000 |  |  |  |  |  |  |  |  |  |  |  |
| Deang | 0.000 | 0.013 | 0.049 | 0.001 | 0.206 |  |  |  |  |  |  |  |  |  |  |
| Khmus | 0.000 | 0.000 | 0.000 | 0.000 | 0.000 | 0.000 |  |  |  |  |  |  |  |  |  |
| Tibetean(YN) | 0.096 | 0.473 | 0.048 | 0.065 | 0.000 | 0.000 | 0.000 |  |  |  |  |  |  |  |  |
| Tibetan(TB) | 0.658 | 0.659 | 0.219 | 0.417 | 0.000 | 0.002 | 0.000 | 0.136 |  |  |  |  |  |  |  |
| Tibetan(QH) | 0.054 | 0.361 | 0.029 | 0.277 | 0.000 | 0.001 | 0.000 | 0.055 | 0.438 |  |  |  |  |  |  |
| Han(BJ) | 0.000 | 0.000 | 0.000 | 0.000 | 0.000 | 0.000 | 0.000 | 0.360 | 0.000 | 0.000 |  |  |  |  |  |
| Han(XA) | 0.000 | 0.000 | 0.000 | 0.000 | 0.000 | 0.000 | 0.000 | 0.028 | 0.000 | 0.000 | 0.552 |  |  |  |  |
| Han(KM) | 0.000 | 0.000 | 0.000 | 0.000 | 0.000 | 0.000 | 0.000 | 0.004 | 0.000 | 0.000 | 0.049 | 0.620 |  |  |  |
| Han(HK) | 0.007 | 0.019 | 0.015 | 0.008 | 0.000 | 0.000 | 0.000 | 0.123 | 0.003 | 0.000 | 0.288 | 0.149 | 0.083 |  |  |
| Evenki | 0.000 | 0.000 | 0.009 | 0.000 | 0.000 | 0.000 | 0.000 | 0.232 | 0.006 | 0.000 | 0.007 | 0.000 | 0.000 | 0.177 |  |
| Oroqen | 0.000 | 0.000 | 0.000 | 0.000 | 0.000 | 0.000 | 0.000 | 0.034 | 0.000 | 0.000 | 0.001 | 0.000 | 0.000 | 0.008 | 0.023 |

^a^ Details of populations are described in Table 6.

**Supplementary Table 3**. Matrix of genetic distances among Chinese ethnic populations

| population^a^ | Thai(DH) | Jingpo | Achang | Deang | Thai(BN) | Khmus | Thai(YX) | Tibetan(TB) | Tibetan(QH) | Tibetan(YN) | Han(BJ) | Han(XA) | Han(KM) | Evenki | Oroqen |
| --- | --- | --- | --- | --- | --- | --- | --- | --- | --- | --- | --- | --- | --- | --- | --- |
| Thai(DH) |  |  |  |  |  |  |  |  |  |  |  |  |  |  |  |
| Jingpo | 0.050 |  |  |  |  |  |  |  |  |  |  |  |  |  |  |
| Achang | 0.107 | 0.126 |  |  |  |  |  |  |  |  |  |  |  |  |  |
| Deang | 0.090 | 0.090 | 0.064 |  |  |  |  |  |  |  |  |  |  |  |  |
| Thai(BN) | 0.039 | 0.043 | 0.098 | 0.077 |  |  |  |  |  |  |  |  |  |  |  |
| Khmus | 0.139 | 0.151 | 0.200 | 0.137 | 0.168 |  |  |  |  |  |  |  |  |  |  |
| Thai(YX) | 0.066 | 0.070 | 0.105 | 0.073 | 0.074 | 0.143 |  |  |  |  |  |  |  |  |  |
| Tibet(TB) | 0.034 | 0.070 | 0.133 | 0.128 | 0.056 | 0.187 | 0.070 |  |  |  |  |  |  |  |  |
| Tibet(QH) | 0.056 | 0.072 | 0.166 | 0.168 | 0.062 | 0.213 | 0.095 | 0.040 |  |  |  |  |  |  |  |
| Tibet(YN) | 0.051 | 0.073 | 0.117 | 0.131 | 0.059 | 0.223 | 0.078 | 0.030 | 0.053 |  |  |  |  |  |  |
| Han(BJ) | 0.065 | 0.074 | 0.103 | 0.115 | 0.059 | 0.226 | 0.092 | 0.082 | 0.093 | 0.041 |  |  |  |  |  |
| Han(XA) | 0.084 | 0.097 | 0.099 | 0.124 | 0.072 | 0.230 | 0.087 | 0.088 | 0.118 | 0.040 | 0.016 |  |  |  |  |
| Han(KM) | 0.093 | 0.115 | 0.115 | 0.139 | 0.099 | 0.219 | 0.085 | 0.079 | 0.129 | 0.045 | 0.032 | 0.013 |  |  |  |
| Evenki | 0.091 | 0.089 | 0.120 | 0.108 | 0.093 | 0.204 | 0.057 | 0.112 | 0.111 | 0.085 | 0.053 | 0.065 | 0.070 |  |  |
| Oroqens | 0.149 | 0.133 | 0.156 | 0.164 | 0.124 | 0.251 | 0.091 | 0.124 | 0.117 | 0.082 | 0.066 | 0.058 | 0.064 | 0.059 |  |
| Han(Hk) | 0.081 | 0.089 | 0.147 | 0.165 | 0.091 | 0.226 | 0.089 | 0.073 | 0.089 | 0.030 | 0.038 | 0.022 | 0.023 | 0.078 | 0.067 |

^a^ Details of populations are described in Table 6.
